# Mental Health in Residential Healthcare Workers During the COVID-19 Pandemic: The Moderating Role of Selfobject Needs

**DOI:** 10.3389/fpsyt.2021.596618

**Published:** 2021-10-27

**Authors:** Eamonn P. Arble, Sneha Shankar, Steven W. Steinert, Ana M. Daugherty

**Affiliations:** ^1^Department of Psychology, Eastern Michigan University, Ypsilanti, MI, United States; ^2^Department of Psychology, Department of Psychiatry and Behavioral Neurosciences, and Institute of Gerontology, Wayne State University, Detroit, MI, United States

**Keywords:** COVID-19, nursing home, post-traumatic stress disorder, depression, interpersonal functioning

## Abstract

The COVID-19 outbreak has affected healthcare across all levels. Older adults and those with chronic illness are at greatest risk for infection complications and mortality, which presents significant psychological distress for residential healthcare workers. The concept of selfobject needs, consisting of Mirroring, Idealizing, and Twinship, may be relevant in explaining psychological distress. This study seeks to enhance our understanding of the needs of healthcare workers responsible for elderly patients and evaluate the role of psychosocial support through selfobject needs to mitigate the effects of trauma during the pandemic. Participants (*N* = 103) employed in residential healthcare facilities in the metropolitan Detroit, MI (USA) region completed an online survey during the peak initial infection. Assessments included standardized measures of trauma-related symptoms, depression, anxiety, and general distress symptoms, as well as a validated measure of selfobject needs. Residential healthcare workers reported mental health symptoms across domains, including clinical elevations in symptoms of trauma, depression, and anxiety. Selfobject needs and mental health outcomes were positively correlated, indicating that greater unmet relational need was associated with greater severity of symptoms. Greater trauma symptom severity as a proxy index of current experience during the pandemic predicted high depressive symptoms, and greater Mirroring need worsened the effect. These results suggest that interventions targeting selfobject needs, specifically Mirroring, may be effective at mitigating acute mental health symptoms among healthcare workers during a distressing event.

## Introduction

The emergence of the COVID-19 pandemic in the United States created a crisis across all levels of healthcare. In the initial infection waves, the crisis was defined by the rapid spread of the severe acute respiratory syndrome coronavirus-2 (SARS-CoV-2), and the limitations of medical knowledge to treat and contain the illness at the time. In response to increasing patient demand, healthcare employees contended with extended work hours, fear for their own personal safety, anxiety for the safety of co-workers and loved ones, and ongoing exposure to the death and suffering of patients (1, 2). These concerns were amplified in residential healthcare, consisting of nursing homes, assisted living communities, and in-home healthcare that provide long-term and post-acute recovery care for some of the highest-risk populations, including older adults and those with chronic medical conditions (1, 3). These challenges faced by providers within residential healthcare were compounded by insufficient staff, depleted supplies, transfer of new patients to these services, and patients whose physical and psychological welfare were deteriorating due to the crisis (4). Healthcare workers in these settings experienced primary and secondary trauma (5) with significant emotional consequences, including acute symptoms of post-traumatic stress disorder (PTSD), depression, and general distress (6, 7). Similar patterns were observed in the 2003 SARS-CoV-1 outbreak (8), where it was found that social isolation and attachment insecurity mediated the effects of trauma exposure on greater mental health symptom severity (9). The current study aimed to evaluate the role of selfobject needs, a personality construct that is reflective of the psychological need for interpersonal connection, in modifying anxiety and depression symptom severity related to trauma symptoms in residential healthcare workers during the COVID-19 pandemic.

While emotional distress and secondary trauma in palliative care is typical for residential healthcare workers (10, 11), the experience during the COVID-19 pandemic was expected to be more severe and also have new sources of distress. The increased exposure to grief and loss, the influx of new patients requiring post-acute recovery care, new safety procedures in place to mitigate transmission, and organizational shortages in necessary supplies were new and defining features of residential healthcare worker experiences during the COVID-19 pandemic (1). Furthermore, these challenges were exacerbated by the need for quarantine and social distancing outside of working hours, which can be reflected in feeling a lack of emotional support. The disruption of personal and professional support challenged trust in leadership across professional and social contexts, including concerns about lack of adequate training and material resources (12). At the time, activities permissible during time outside of work were to some extent controlled by government officials, further compounding concerns with organizational policies, broadly defined. In this sense, all healthcare workers were vulnerable to psychological distress during the COVID-19 pandemic and residential healthcare employees were uniquely experiencing an intersection of challenges with the chronically ill and elderly, with heightened isolation protocols.

The broad disruption to social and emotional support created a vulnerability for residential healthcare workers to develop poor mental health following the increase in stress and trauma during the pandemic. Developing a nuanced understanding of the experiences of healthcare workers, especially those in residential facilities that were strongly impacted by COVID-19, is crucial for informing policies to support their personal well-being. Development of clinical mental health disorders is not inevitable after traumatic experiences, and identifying factors that ameliorate acute symptoms may be useful to promote resiliency in the frontline healthcare workforce.

Selfobject needs, as proposed by Heinz Kohut's theory of self psychology (13–16), is one theoretical model that can account for the experience of distress and other psychological symptoms, as well as the relational and personality features that are predictive of healthcare workers' responses to distress and trauma. This theory has been employed in a variety of clinical contexts (17), has proven relevant for occupational functioning (18), contributes to emotional resilience (19), and can be reliably measured (20). In brief, the theory advanced by Kohut proposes that an individual's ability to navigate emotional challenges will in large part be dependent upon the extent to which they have had, and continue to have, specific relational needs met. In essence, an individual whose core relational needs are being met is able to develop and maintain a healthy sense of self, which encapsulates aspects of purpose, self-worth, and belonging. Conversely, individuals whose needs are not being met may experience a diminished sense of self, which can lead to a variety of clinical difficulties, as well as an inability to remain emotionally regulated in the face of internal and external challenges.

Kohut proposed the existence of three selfobject needs: Mirroring, Idealizing, and Twinship (13–16). Mirroring refers to receiving recognition for one's contributions and value. An in-home healthcare worker whose family members empathize with their struggles and praise them for their bravery and self-sacrifice may experience the sense of having their value being “mirrored.” As the need for Mirroring reflects that people need to have their own value recognized, Kohut proposed that individuals also seek to connect themselves with others whose value they recognize. An individual who experiences someone in their life as admirable and worthy of emulation may experience the benefit of Idealizing. For example, a nurse who admires their supervisor's ability to maintain a sense of calm while capably performing their duties may feel that their connection with this supervisor enhances their own self-worth, and that nurse may derive a sense of striving and purpose as a result of this connection. Finally, the selfobject need of Twinship refers to experiencing a sense of belonging and participation, a recognition of oneself in someone else. For example, a social worker conversing with a colleague, seeing another person who truly understands their struggles and motivations, may experience a sense of connection and “alikeness.” This experience could be understood as a feeling of Twinship.

According to Kohut, having these needs met over the course of development results in a healthy sense of self, a kind of self-cohesion that would allow the developing individual to withstand the emotional and relational challenges facing them in their life. However, a fundamental component of the self psychology articulated by Kohut was the premise that the need for selfobject responsiveness is never outgrown (14). People require the interaction of others to maintain their sense of self, and lack of such experiences can prove to be harmful at any stage of life. Much as the well-fed individual can comfortably miss a meal but nonetheless requires nourishment, an individual whose selfobject needs have historically been met is more likely to demonstrate emotional resilience, but is still vulnerable to their absence or insufficiency. Of particular relevance to the current pandemic, selfobject need increases in times of duress and transition, such that even individuals with strong and positive relational histories may find themselves overwhelmed in the face of extraordinary demands and isolation (21).

The concept of selfobject needs has received theoretical and empirical consideration for some time (21, 22), with recent research highlighting its utility when considering the experience of potentially traumatic events (23, 24). Contemporary theorists and clinicians have continued to draw upon and elaborate on the selfobject concept, identifying new pathways for its use in both assessment and intervention (25, 26). It has been well-documented that the ability to draw upon social relationships is an important coping strategy that can effectively alter stress responses on an emotional and physiological level (27, 28). However, the ability to utilize relational resources may in part be dependent upon an individual's view of self (29, 30), and some social networks may be unable to provide the needed support, the lack of which may ultimately result in the development of symptoms such as loneliness and depression (31–33).

This sequence of encountering a difficult experience, followed by social disruption and intense negative emotions, fits well-within the self psychology framework (34). In this understanding, trauma is conceptualized *via* the external event *and* the subjective and introspective meaning applied to the event (35). According to Kohut, the experience of a potentially traumatic event can provoke a rupture in selfobject connection, such that the individual no longer inhabits a world where they feel safe, understood, and valued (36). As the individual is left to grapple with the traumatic event and the resulting disruption to their view of themselves and the world, their sense of self can begin to fragment. Unmet selfobject needs, in turn, leaves them vulnerable to emotional disruptions beyond their initial trauma reaction, such as depression, loneliness, and pain (34, 37).

Given the ongoing stress, potential for traumatic experience, and social isolation caused by quarantine, it seems that there are numerous parallels between the theory of self psychology and the stressors facing healthcare workers during this crisis. However, the relevance of selfobject needs for residential healthcare workers requires empirical study. What is particularly needed is an investigation of the role of the specific selfobject needs within the context of the psychological symptoms experienced by these healthcare workers, which may help to identify interventions to promote psychological resiliency.

The current study addresses this in a sample of residential healthcare workers in the metropolitan Detroit, Michigan (USA) region during the initial peak COVID-19 infection. At the time of the study, Michigan was among the top 10 ranked states in the number of confirmed cases and deaths (38), with the majority presenting in the metropolitan Detroit region, and approximately a third of deaths had been reported from residential healthcare facilities (39). Self-reported trauma symptoms were considered a proxy for the healthcare employee's current experience of distress or trauma during the COVID-19 pandemic, as well as reported severity of depression, anxiety, and perceived stress with established clinical measures. We tested the following hypotheses. First, greater selfobject need correlates with higher reports of symptoms of trauma, depression and anxiety, and greater perceived stress. Second, greater selfobject need will increase the positive association of trauma symptoms with depression and anxiety symptom severity, consistent with an exacerbated effect of trauma on mental health.

## Materials and Methods

### Participants

Employees in residential healthcare settings in the metropolitan Detroit region were surveyed through an anonymous, online platform from April 29 to May 14, 2020. The sample was recruited through email addressed to employees and directors of local residential healthcare agencies including assisted living communities, nursing homes, and in-home care. Potential participants were contacted through an email listserv, which local healthcare workers had joined to learn about research and education opportunities. Administrative staff and directors of local agencies were also contacted by email and were asked to distribute the survey information among staff. Of the 148 individuals who initiated the survey, 103 completed responses to all survey items; data were missing at random [Little's χ${}_{\left(16\right)}^{2}$ = 14.78, *p* = 0.54] and the sample with complete data were included in the current report. The sample of 103 employees (90.3% female) were on average middle-age (*M* = 52.32, *SD* = 11.22; range = 24–79 years). The sample was predominantly non-Hispanic White (72.8%) and Black (12.6%), all other racial and ethnic groups included few respondents (total 7.8%); 6.8% of the sample declined to report. Consistent with a prior published report of traumatic experience of healthcare workers in a previous SARS pandemic that was associated with social isolation and attachment insecurity (9), the current study was planned for a minimum target sample size of 100 to power (>0.80) tests to detect moderate effect sizes (*f* ^2^ ≥ 0.08) to significance (α = 0.05). The study was approved by Institutional Review Boards; all participants provided consent by initiating the online survey after reading the study information.

### Measures

#### Arble Estimate of Selfobject Pursuits

The Arble Estimate of Selfobject Pursuits (AESOP) is a 31-item measure of selfobject needs. Separate scores are generated for each of the three proposed selfobject needs: Mirroring, Twinship, and Idealizing. All items are scored on a 1 (“Not at all true of me”) to 7 (“Very true of me”) scale, with higher scores reflecting greater amounts of unmet needs. Sample items include: “I feel that people do not appreciate the struggles I've had to face.” The AESOP was developed by clinicians and researchers operating within a self psychology framework (20). In its initial study, a sample of 686 participants completed the measure, and an exploratory factor analysis was conducted (20). A three-factor solution was identified, corresponding to the three identified selfobject needs. In a second study, a sample of 672 respondents completed the measure, as well as a number of related measures (20). The three-factor structure that was identified with the EFA from the previous study was confirmed using a latent modeling technique. The items were entered into a confirmatory factor analysis (CFA); model fit was evaluated with a compendium of accepted fit indices, all indicating adequate-strong fit. This second analysis is critical, as the AESOP's structure has been identified in both exploratory and confirmatory analyses, offering it a unique strength as compared to other measures of selfobject needs, such as the SONI (22). The use of both exploratory and confirmatory analyses is highly beneficial for measurement validation, as due to constraints within the modeling process, confirmatory and exploratory analyses can produce discrepant results and do not share the same assumptions (40, 41). The SONI, while generally demonstrating theoretically consistent patterns of relationships with the AESOP, has not fared as well in subsequent psychometric analyses, with its proposed factor structure proving somewhat tenuous (20, 42).

Finally, cluster and discriminant function analyses provide strong evidence of the AESOP's convergent and discriminant validity (20). AESOP scale scores in the sample had good internal consistency: Cronbach's α = 0.93, 0.82, and 0.85, Mirroring, Idealizing, and Twinship, respectively, which is in agreement with the prior report of a community sample (20). Means and standard deviations of the published normative sample of 672 respondents (20) were used to calculate standardized scores to evaluate the similarity of responses collected during the pandemic to typical responses: standardized scores equal to zero indicate responses equal to the normative sample mean and variation is scaled to the standard deviation. All hypotheses tests were completed with the sample scale scores.

#### Depression and Anxiety Stress Scales-21

The Depression and Anxiety Stress Scales-21 (DASS-21) is a 21-item measure of depression, anxiety, and stress (43). Separate scores are generated for each of the respective clinical constructs. All items are scored on a 0 (“Never”) to 3 (“Almost Always”) scale, with higher scores reflecting greater levels of symptoms. Sample items include: “I felt that I had nothing to look forward to.” The measure's reliability and validity have been established in numerous studies, and it has been utilized in recent research among healthcare workers (44). Depression and Anxiety Stress Scales-21 scale scores had good internal consistency, Cronbach's α = 0.91, 0.83, and 0.91 for depression, anxiety and stress scales, respectively, similar to other reports of non-clinical community samples (45, 46).

#### PTSD Checklist for DSM-5

The PTSD Checklist for DSM-5 (PCL-5) is a 20-item measure of PTSD symptoms (47). Items are rated on a 0 (“Not at all”) to 4 (“Extremely”) scale, with higher scores reflecting greater levels of trauma symptoms. Sample items include: “Being ‘superalert' or watchful or on guard?” The PCL-5 is a popular measure of PTSD symptoms and has been found to be a reliable and valid instrument (48), and had high internal consistency in the present sample (Cronbach's α = 0.95), similar to another report of trauma-exposed, community sample (49). The PCL-5 is sometimes supplemented with measures of Criterion A, the PTSD diagnostic criteria requiring that the individual be exposed to a traumatic event. These supplemental measures can take the form of checklists (e.g., a list of potentially traumatic events that the respondent can endorse the experience of) or free-from responses where the individual reports and describes the traumatic event they experienced. These supplements were not administered in this survey, meaning that while the present PCL-5 score can provide a measure of trauma symptoms, they cannot be interpreted as a diagnosis.

### Statistical Analysis

Prior to hypothesis testing, data were screened for normality of univariate distributions, as well as cases that were outliers to the sample. Distributions of depression, anxiety, and perceived stress scores were positively skewed; because the scale scores have clinical interpretations that would be lost if a transformation were applied, hypothesis tests were selected that provide robust estimates for non-normal distributions and original scale scores were used in all analyses. Two cases were identified as univariate (*z*-score > |3.29|) or multivariate outliers (Mahalanobis distance, critical χ^2^ = 24.322, α = 0.001). Regression estimates are vulnerable to bias from extreme or leverage cases; however, maintaining the complete eligible sample improves the external validity of the estimates. Therefore, all analyses were conducted with complete data (*N* = 103) and hypothesis tests were repeated after removing outlier cases to confirm negligible bias in the estimates.

Hypothesis 1 was tested with Spearman correlations (ρ*)*, which do not require a normal distribution for valid estimates (50), including bias-corrected bootstrapped 95% confidence intervals [5,000 draws (51); BS 95% CI], which if not overlapping with zero would provide further support for the hypothesized relation. Possible differences in the magnitude of the correlation with mental health outcomes between selfobject needs were tested with Steiger Z (52). To account for the multiple comparisons made, all significance testing was adjusted for false discovery rate (FDR-adjusted *q*-value) (53).

Hypothesis 2 of selfobject need as a moderator of the effect of trauma symptoms on depression and anxiety symptom severity was tested in a two-level repeated measures general linear model with multivariate estimates, which does not require normal univariate data distributions for valid estimates in large samples (54) and takes into account the correlations among scale responses for depression and anxiety. The model included trauma symptoms, scale scores for Mirroring, Idealizing, and Twinship, and two-way interactions with each, as independent variables predicting depression and anxiety as a two-level repeated measurement (Depression/Anxiety). To alleviate expected multicollinearity among predictors and to allow comparisons across sub-scales, the selfobject need measures were converted to sample *z*-scores prior to model estimation. All multivariate *F*-tests are reported with standardized effect sizes (η_*p*_^2^). Significance of the interaction term (α = 0.05) and 95% CI not overlapping zero were accepted as evidence of moderation; omnibus effects were decomposed with univariate, hierarchical regression procedures.

## Results

### Sample Description

The survey participants (*N* = 103) reported 1–46 years of experience working in residential healthcare (*M* = 20.35, *SD* = 12.91). Reported job titles and descriptions indicated a wide representation of roles within the setting. The majority of participants were social workers (33.0%), followed by nurses (16.5%), agency directors or owners (17.5%), occupational therapists (6.8%), specialty consultants or care coordinators (5.8%), administration or technical staff (5.8%), psychologists (3.9%), nurse assistants (3.9%), and physical therapists (1.9%). Other respondents reported a role as doctor, sales associate, and human resources.

Scores across the measures of PTSD (range = 0–62; *M* = 20.56, *SD* = 16.76), depression (range = 0–20; *M* = 4.27, *SD* = 4.79), anxiety (range = 0–18; *M* = 3.29, *SD* = 3.84), and perceived stress (range = 0–18; *M* = 6.14, *SD* = 5.02) fell, on average, within the normal range, though 35% of individuals reported clinical elevations.

To compare the level of reported selfobject need to a normative sample of 672 respondents, we examined z-scores calculated from the published means and standard deviations (20): Mirroring (*M* = −0.46, *SD* = 1.07; BS 95% CI: −0.66/−0.26), Twinship (*M* = −0.65, *SD* = 1.18; BS 95% CI: −0.85/−0.43), and Idealizing (*M* = −0.93, *SD* = 1.26; BS 95% CI: −1.17/−0.70). Based on the BS 95% CI, the average response on all scales did not overlap with zero and indicated that responses were lower than the normative reference. Therefore, reported responses in this sample are within the normal range of reported selfobject needs observed in the reported community sample (20).

### Hypothesis 1: Correlation of Selfobject Need With Mental Health

Bivariate correlations between selfobject needs and self-report mental health outcomes are reported in Table 1. As expected, responses to Mirroring, Idealizing, and Twinship scales were positively correlated. Across sub-scales, higher selfobject need positively correlated with greater experience of depression, anxiety and trauma symptoms, and perceived stress. Comparing the magnitude of the correlation coefficients, Mirroring was more strongly correlated with symptom severity than Idealizing across all symptom domains (all *z* > 3.47, *q* < 0.01), and more strongly correlated with trauma and anxiety symptoms severity as compared to Twinship (both *z* > 2.90, *q* < 0.01). Mirroring was also more strongly associated with perceived stress as compared to Idealizing (*z* = 2.25, *q* = 0.04) but not Twinship (*z* = 1.35, *q* = 0.21). Twinship was more strongly correlated with depression as compared to Idealizing (*z* = −2.46, *q* = 0.03), but each were similarly correlated with anxiety (*z* = 0.15, *q* = 0.88) and trauma symptoms (*z* = −0.93, *q* = 0.38). In sum, higher selfobject need was associated with worse reports of depression, anxiety, trauma, and distress symptoms, and the relation was, in general, stronger with Mirroring as compared to Idealizing and Twinship.

**Table 1:** Bivariate correlations among selfobject needs and self-report mental health among residential healthcare workers.

		**1**	**2**	**3**	**4**	**5**	**6**
1	Mirroring	1.00					
2	Idealizing	0.60 (0.44/0.72)	1.00				
3	Twinship	0.53 (0.37/0.67)	0.73 (0.61/0.81)	1.00			
4	PTSD	0.68 (0.57/0.76)	0.41 (0.23/0.55)	0.47 (0.30/0.61)	1.00		
5	Depression	0.64 (0.49/0.76)	0.33 (0.14/0.50)	0.49 (0.31/0.64)	0.77 (0.67/0.84)	1.00	
6	Anxiety	0.59 (0.46/0.70)	0.33 (0.14/0.49)	0.32 (0.13/0.50)	0.77 (0.68/0.84)	0.65 (0.50/0.77)	1.00
7	Stress	0.62 (0.49/0.73)	0.46 (0.29/0.60)	0.52 (0.37/0.64)	0.84 (0.78/0.89)	0.74 (0.65/0.81)	0.66 (0.54/0.75)

*Bivariate Spearman correlations (ρ) are reported with bias-corrected bootstrapped 95% confidence intervals (lower limit/upper limit). All correlations were significant after FDR correction, q < 0.001*.

### Hypothesis 2: Selfobject Need Moderates the Effects of Trauma Symptoms on Depression and Anxiety

We hypothesized that high reports of trauma symptoms would be associated with high severity of depression and anxiety symptoms, and that greater selfobject need would worsen these relations. Higher trauma symptom severity significantly predicted mental health outcomes [*F*_(1, 97)_ = 103.36, *p* < 0.001; η_*p*_^2^ = 0.52]. The effect of trauma symptom severity was moderated by Mirroring, and the magnitude of this complex effect differed between anxiety and depression (Depression/Anxiety × Trauma × Mirroring): *F*_(1, 97)_ = 4.74, *p* = 0.03; η_*p*_^2^ = 0.05. There was no evidence of Twinship [*F*_(1, 95)_ = 1.18, *p* = 0.28] or Idealizing [*F*_(1, 95)_ = 0.002, *p* = 0.96] moderating the effect of trauma symptoms on mental health outcomes. Therefore, these interaction terms were removed from the model.

**Figure 1:**
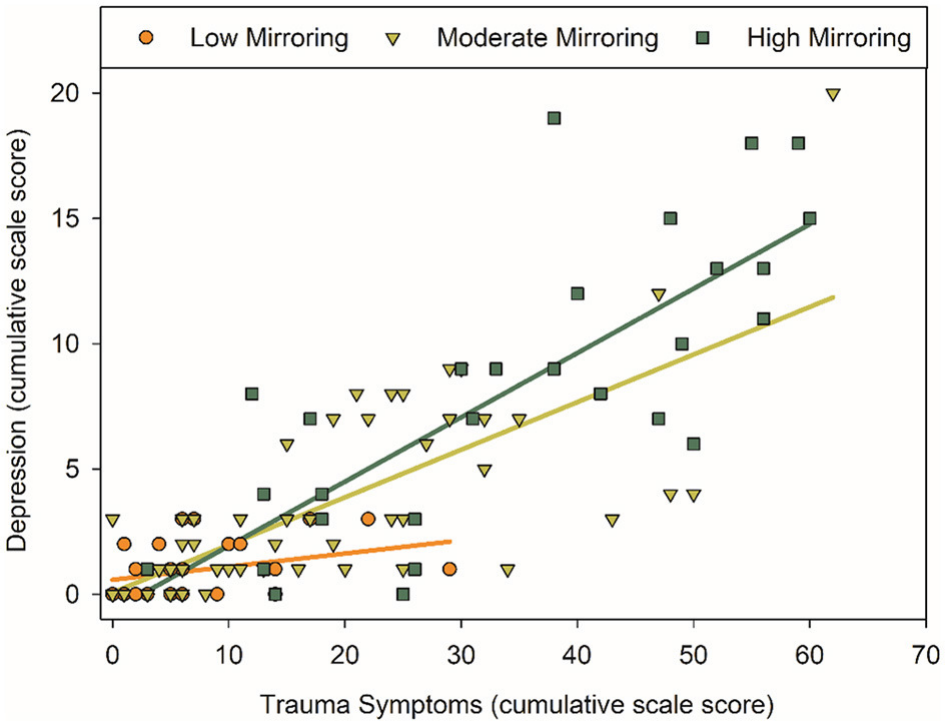
The association between PTSD and depression symptoms was moderated by Mirroring need. All analyses treated cumulative scale scores as continuous variables; for the purpose of illustration, Mirroring need is depicted at low (≥25^th^ percentile), moderate, and high (≥75^th^ percentile) levels as observed in the sample. Higher PTSD symptoms were positively correlated with higher depression symptoms. This association was more strongly positive at moderate and high levels of Mirroring (*b* = 0.003, *p* = 0.01, Δ*R*^2^ = 0.02). Depression scores 11 and higher indicate “severe” to “extremely severe” levels; notably individuals who reported lower Mirroring need did not pass this threshold despite reporting elevated PTSD symptoms.

To further examine the complex interaction between trauma symptoms and Mirroring in predicting depression and anxiety differentially, we evaluated univariate regressions in a *post-hoc* analysis. Mirroring significantly moderated the relation between trauma symptoms and depression (Trauma × Mirroring): *b* = 0.003, *p* = 0.01; 95% CI: 0.001/0.005; Δ*R*^2^ = 0.02. Individuals with higher Mirroring need demonstrated a stronger positive relation between trauma symptoms and depression symptoms (Figure 1). Depression scores 11 and higher indicate “severe” to “extremely severe” levels; notably individuals who reported lower Mirroring need did not pass this threshold despite reporting elevated trauma symptoms. Trauma symptoms and Mirroring, together, accounted for a large proportion of variance in reported depression symptoms (model *R*^2^ = 0.69, *p* < 0.001). There was no evidence of Mirroring moderating the relation between trauma symptoms and anxiety (*b* = 0.00, *p* = 0.71; 95% CI: −0.002/0.002; Δ*R*^2^ = 0.001), but high Mirroring need and trauma symptoms accounted for approximately half of the variance in anxiety symptom severity (model *R*^2^ = 0.59, *p* < 0.001). Repeating the analysis after removing two cases that were statistical outliers replicated the same pattern of results: Mirroring moderated the relation between trauma symptoms and depression (*b* = 0.003, *p* < 0.01; 95% CI: 0.001/0.004; Δ*R*^2^ = 0.02) but not anxiety (*b* = 0.00, *p* = 0.88; 95% CI: −0.002/0.002; Δ*R*^2^ = 0.00). Taken together, trauma symptoms was the strongest, unique predictor of depression and anxiety symptom severity, and higher Mirroring need exacerbated the effect on depression.

## Discussion

We report on a diverse sample of residential healthcare workers who were active during the initial peak COVID-19 infection in a strongly impacted metropolitan region. Participants reported a change in daily activities from COVID-19, as well as elevations in distress, trauma, depression, and anxiety symptoms. Selfobject needs presented as a strong correlate of these clinical symptoms across all domains. Although a proportion of individuals reported clinical elevations, the average responses indicated many symptoms fell within normal range. This evidence that is consistent with emotional resiliency, despite disruption to daily activity and distress, is promising. In the current report we explored a possible source of resiliency in the correlation of selfobject needs with symptom profiles. High Mirroring need worsened the association between trauma symptoms and depression severity; and consistent with resiliency, those with low Mirroring need did not report clinically severe depression symptoms despite experiencing trauma symptoms.

As understood by Kohut, unmet selfobject needs disrupt core aspects of a person's internal world, thereby leaving them vulnerable to a variety of deleterious outcomes. In line with this theory, each of the three selfobject needs correlated with all of the clinical outcomes assessed. As would be theoretically expected, individuals reporting higher levels of unmet selfobject needs were more likely to report greater levels of trauma symptoms, depression, anxiety, and stress. In many respects, identifying that a person is in a state of need (i.e., having their selfobject needs unfulfilled) may be a powerful indication that they are in danger of experiencing a variety of associated psychological concerns. Given the challenges facing those working in residential healthcare, the field would do well to pay careful attention to these indicators.

Witnessing patient illness and death, fears of contracting COVID-19, and the strain of quarantine all leave healthcare workers in residential healthcare vulnerable to traumatic exposure and resulting symptoms. High trauma symptom severity and traumatic experiences increase the risk to develop major depressive and anxiety disorders (55), and comorbid symptom profiles have been reported in frontline healthcare workers during traumatic events (56, 57). However, most people undergoing a traumatic event are unlikely to develop lasting symptoms of trauma (58). We were thus curious as to the role that selfobject needs might play in moderating the association between experience of trauma symptoms and the symptoms of other mood disorders, particularly given the connection between PTSD and emotional reactivity (59). Although each of the three selfobject needs positively correlated with mental health symptom severity, the correlation with Mirroring need was in general stronger than that with Idealizing and Twinship needs. In community samples, Mirroring has been previously found to be a stronger correlate of self-reported mental health symptoms than the other selfobject needs, and the effect is general and does not discriminate between symptom domains (20). This may indicate that Mirroring is more easily accessible among community samples due to its connection with self-esteem (13–16), whereas Twinship and Idealizing needs differentiate clinical elevations in personality and psychological symptoms (20) that were not observed in this sample. Mirroring emerged as particularly important in understanding the relation between trauma symptoms and depression—among individuals who reported higher levels of unmet needs for Mirroring, the association between trauma symptoms and depression was strengthened. When considering the recommended cutoffs of the depression scale, reports >11 would indicate clinically severe symptoms, and notably healthcare workers who reported low Mirroring need did not have severe elevations in depression symptoms despite experiencing trauma symptoms. Mirroring need, trauma symptoms and the interaction together accounted for 69% of variance in depression symptom severity. Mirroring need and trauma symptoms also accounted for 59% of variance in anxiety symptom severity, but there was no evidence of a moderating effect. The differential effects between depression and anxiety symptoms may reflect the circumstance of the data collection. Residential healthcare workers were surveyed during the initial infection wave, when the nature of COVID-19 disease transmission was poorly understood, rapid testing was not widely available, policies were rapidly changing, and a vaccine was a distant promise. In these circumstances, acute sub-clinical elevation in anxiety may be typical and correlated with but not modified by selfobject need, whereas acute depression symptoms plausibly can be modified in that context. Although speculative, this suggests the intriguing possibility that by satisfying the need for Mirroring, some of the negative consequences of trauma exposure could be blunted, even during acute responses to trauma before chronic disorders arise.

Mirroring consists of receiving recognition for one's efforts thereby resulting in the “mirroring” of one's value. Mirroring is critical to the development and maintenance of self-esteem (13–16). From the present study's results, it appears that meeting one's needs for Mirroring can increase resiliency to negative events, an important benefit during the ongoing pandemic. Fortunately, the idea of Mirroring as a universal human need that can be fulfilled by people in a healthcare professional's life makes it an excellent target for intervention. Whereas, some interventions may be developed for specific occupations or diagnoses, an emphasis on Mirroring should prove beneficial for all the diverse professionals engaged in residential healthcare. And the ability to provide mirroring to someone does not require special station or expert training; a willingness to empathetically understand the struggles of healthcare professionals and expressing appreciation for their contributions may be sufficient to provide a significant amount of emotional assistance.

Consistent with the present results, previous research has identified that recognition significantly influences stress levels in healthcare professionals (60). Following the previous SARS-CoV-1 pandemic, preventative strategies at an organizational level were suggested to aid frontline healthcare workers to adequately cope with traumatic experiences, and in particular, psychosocial support was identified as an effective tool (9). The literature thus appears to support Kohut's core assertion that the quality of one's relationships, both longstanding and recent, predict the length and severity of symptoms following trauma or adversity (29, 31).

For healthcare systems and employers who identify that their providers are struggling socially or emotionally, there may be some utility in organizational programs focused on building and fostering personal and professional relationships. This is particularly advisable in times of strain such as the present outbreak; due to quarantine, healthcare workers may find themselves isolated from the very support that might be beneficial. Creating a supportive space with time to reflect and evaluate emotional experiences are key aspects of empathic listening that have been shown to ameliorate psychological symptoms, compassion fatigue, and occupational burn-out among nurses, social workers and psychologists (61, 62). There are some examples of organization-level interventions around selfobject needs in clinical care of the elderly (63), and a similar structured approach may be useful when supporting residential healthcare workers. Furthermore, there are available resources to help utilize self psychology principles in a group context (64). Future studies of intervention designs and mental health outcomes will be necessary to implement meaningful and feasible organization-based programs. Facilitating meaningful social connection, directly providing recognition and expressing appreciation for individual healthcare worker's value, and being mindful of the well-being of people employed in residential healthcare, all appear as practical and important tools for our healthcare systems to utilize.

### Limitations

Although this study provides valuable information for the applicability of selfobject needs in understanding and reducing psychological symptoms among healthcare workers, it is not without limitations. The main limitation involved the circumscribed nature of the present assessment and its reliance on self-report questionnaires, which are subject to issues concerning accuracy and self-presentation biases, particularly in the case of selfobject needs. Given that selfobject responsiveness is key to the self psychology conceptualization of psychopathology, considerable theoretical consideration has been given to understanding the experiences of those whose developmental histories were marked by a lack or disruption of these needs. Kohut noted that a child whose pursuit of selfobject needs is characterized by pain and rejection may develop a defensive strategy where this core need is rejected and denied (65). For these individuals, the pursuit of selfobject needs has proven so painful and unsuccessful, they deny the need for pursuit altogether. Unfortunately, this denial cannot provide deep and longstanding emotional fulfillment. In a self-report measure such as the AESOP, an individual using this defensive style can simply deny their experiences, meaning that their state of emotional needs could be undetected. It is unclear if a more thorough assessment (e.g., using clinical interviews) would yield similar results. A more comprehensive assessment, including a view of the participants' relational/developmental history, could better illuminate some of the theoretical concepts being explored.

Second, the present results could only consider symptom totals, as opposed to diagnostic categories. A single self-report measure cannot diagnose a condition such as PTSD, and it would be interesting to consider if the same pattern of correlations would be observed amongst those meeting diagnostic criteria. The inability to offer a diagnosis is particularly relevant in the case of PTSD, as the present assessment did not include a measure of Criterion A (i.e., confirmation of the presence, and nature of, a traumatic experience). Scores on the PCL-5 are driven by symptoms reported in response to “stressful experiences” which may not be traumatic in nature. Even if a traumatic experience was present within the sample, there is no way to confirm that the trauma was in any way related to their occupational experiences. Furthermore, we only evaluate the acute response during the initial peak COVID-19 infection in the region, and symptom severity and chronicity that indicate a clinical disorder may be evident after more time. It is thus important to note that when referencing concepts such as trauma symptoms from PCL-5 responses or selfobject needs, the present research can only provide proxies for these concepts, meaning that interpretation of the observed correlations should be approached conservatively.

Third, several factors related to the source of distress, organizational resources, and occupational role may modify the healthcare worker's experience of trauma during the pandemic. This convenience sample included participants in a multitude of roles, the majority of whom were nurses and social workers, and a small number in administrative roles. Based on sweeping policy changes and public health initiatives (1), any employee in a residential healthcare facility is expected to experience disruption and anxiety for disease exposure, but not all employees will have direct experiences with patient care. Indeed, sources of distress and trauma related to organization resources, such as personal protective equipment or inadequate staffing, is expected to differentially affect frontline healthcare more than administrative staff working in the same environment. The available sample was insufficient to test the hypothesis of occupational role moderating psychological symptom severity, its interaction with selfobject need, or different sources of distress. Similarly, the convenience sample was generally consistent with the demographics reported of regional residential healthcare workers, but Black healthcare workers were under-represented. Social and behavioral determinants of mental health contribute to increased experience of trauma and its consequences for Black adults (66) and we cannot evaluate possible racial and ethnic differences in the current sample. The limited data collection through anonymous survey also precluded analyses of selection bias related to missing data responses. Future studies should endeavor to not only improve sample diversity, but also consider mixed methods approaches to systematically evaluate the complete experience of the residential healthcare workforce across occupations.

## Conclusion

In summary, we report on a diverse sample of healthcare workers in residential healthcare settings throughout the metropolitan Detroit region during the peak of the initial COVID-19 infection. Employees in these settings experienced a substantial disruption to daily activities. Although some individuals reported elevations in trauma symptoms, depression, and anxiety symptoms, and heightened stress, the average response on these scales fell within normal range. Selfobject needs presented as a strong correlate of symptom severity across the domains, and greater Mirroring need, in particular, exacerbated the relation between the experience of trauma symptoms and depression. Emphasizing the importance of relational needs such as Mirroring for healthcare workers in residential healthcare is a feasible approach to promote resiliency in mental health following traumatic experiences during the COVID-19 pandemic and after.

## Data Availability Statement

The raw data supporting the conclusions of this article will be made available by the authors, without undue reservation.

## Ethics Statement

The studies involving human participants were reviewed and approved by Eastern Michigan University IRB. The patients/participants provided their written informed consent to participate in this study.

## Author Contributions

EA, SS, SWS, and AD all contributed to the study's conceptualization and the writing of the manuscript. EA and AD conducted data collection and analysis. All authors contributed to the article and approved the submitted version.

## Conflict of Interest

The authors declare that the research was conducted in the absence of any commercial or financial relationships that could be construed as a potential conflict of interest.

## Publisher's Note

All claims expressed in this article are solely those of the authors and do not necessarily represent those of their affiliated organizations, or those of the publisher, the editors and the reviewers. Any product that may be evaluated in this article, or claim that may be made by its manufacturer, is not guaranteed or endorsed by the publisher.
